# In-Depth Analysis of the Mechanism of Astaxanthin Succinate Diester in Reducing Ulcerative Colitis in C57BL/6J Mice Based on Microbiota Informatics

**DOI:** 10.3390/molecules28186513

**Published:** 2023-09-08

**Authors:** Xing Qiao, Qun Gao, Lu Yang, Xiaoxu Wang, Zhigao Wang, Zhaojie Li, Jie Xu, Changhu Xue

**Affiliations:** 1 College of Food Science and Engineering, Ocean University of China, Qingdao 266003, China; qiaoxing@stu.ouc.edu.cn (X.Q.); ixkx736@163.com (Q.G.); yanglusp@163.com (L.Y.); wangxx0416@163.com (X.W.); wzg940518@163.com (Z.W.); lizhaojie@ouc.edu.cn (Z.L.); xuech@ouc.edu.cn (C.X.); 2 College of Food Science and Technology, Henan University of Technology, Zhengzhou 450001, China

**Keywords:** astaxanthin succinate diester, ulcerative colitis, intestinal microbiota, dextran sulfate sodium (DSS), pro-inflammatory cytokines

## Abstract

This paper aims to explore the effect and mechanism of water-soluble astaxanthin succinate diester (Asta-SD) on ulcerative colitis (UC) induced by dextran sodium sulfate in zebrafish and C57BL/6J mice. Asta-SD was synthesized with hydrophilic fatty acid succinic anhydride and the hydroxyl groups at the ends of F-Asta were synthesized by esterifying. Through the construction of a zebrafish intestinal inflammation model, it was found that Asta-SD could effectively reduce the levels of ROS and increase the number of healthy intestinal lysosomes in zebrafish. After continuous gavage of Asta-SD for seven days, the body weight, disease activity index, colonic length, colonic histopathology, expression of inflammatory factors, and intestinal flora of the mice were measured. The results showed that Asta-SD could significantly alleviate weight loss and colonic shrinkage, as well as reducing pro-inflammatory cytokines and recess injury in UC mice. The 16S rRNA gene sequencing showed that Asta-SD significantly increased the beneficial bacteria (*Lactobacillus*, *Anaerotruncus*) and decreased the relative abundance of pathogenic bacteria, effectively maintaining intestinal microbiota homeostasis in mice. Based on Pearson analysis, *Bacteroides*, *Parabacteroides*, and *Butyrimionas* were expected to be associated with the significant difference in the expression of inflammatory factors between the UC and the corresponding host. Thus, Asta-SD significantly improves UC and maintains intestinal microbiota homeostasis.

## 1. Introduction

Ulcerative colitis (UC) is a chronic intestinal inflammation accompanied by diarrhea, bloody mucus, weight loss, and colon shortening [1]. Furthermore, its pathogenesis is often related to oxidative stress and immune imbalance caused by the environment, genetics, and intestinal microecology [2,3]. Compared with the healthy population, UC patients are characterized by gut microbiota dysbioses, such as a reduced abundance of probiotics (like *Lactobacillus*) [4] and excessive proliferation of inflammation-related bacteria (like *Oscillospira*) [5]. These microbial disorders contribute to the inflammatory response caused by colonic inflammation. Therefore, intestinal microbiota may be potential therapeutic targets of UC. Of course, the production of pro-inflammatory cytokines (such as TNF-α, IL-1β, and IL-6) will also increase with the occurrence of chronic inflammation [6]. On the other hand, interleukin-10 (IL-10) is a critical immunosuppressive cytokine in UC [7]. The commonly prescribed therapeutic drugs for UC are corticosteroids, aminosalicylic acid, and immunosuppressants [8]. However, drug therapy has a limited therapeutic effect and a range of evident side effects, and patients will develop drug resistance [9,10]. Therefore, developing new therapies (such as natural anti-inflammatory small molecules) with weaker side effects and higher efficacy for UC is essential.

**Figure 1:**
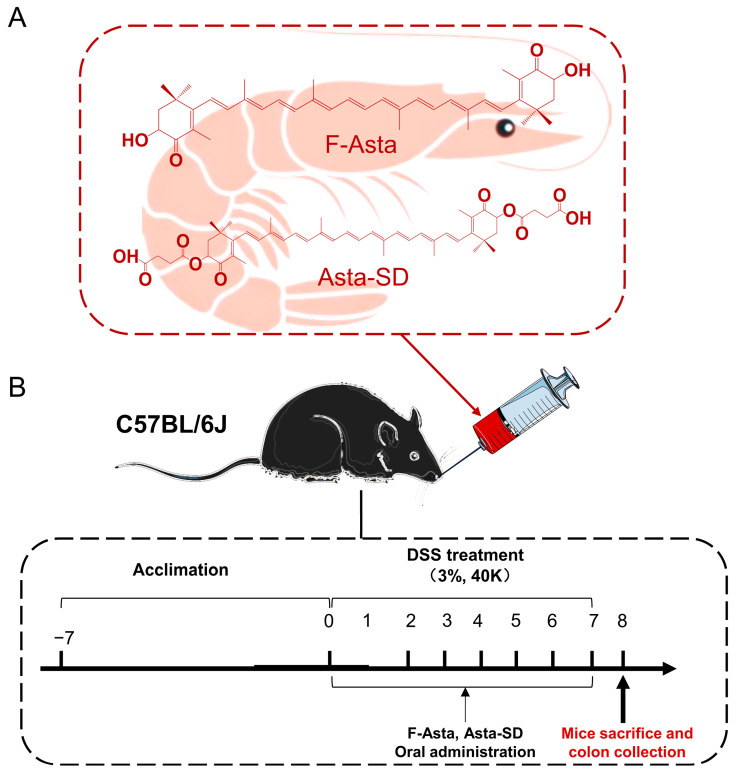
Study design for the whole experiment. The chemical structure of free astaxanthin (F-Asta) and astaxanthin succinate diester (Asta-SD) are presented in panel (**A**). C57BL/6J mice were provided with water or 3% DSS-containing water for 7 d (**B**). On days 0–7, mice were orally administered with PBS or 50 mg kg^−1^ of F-Asta, Asta-SD.

Astaxanthin (Asta) is one of the natural red fat-soluble carotenoids and exists in two forms: free astaxanthin (F-Asta) and astaxanthin esters (Figure 1A). Asta contains a β-ionone ring and a polyunsaturated conjugated double bond system. Thus, it has the ability to quench singlet oxygen and is an excellent scavenger of reactive oxygen species (ROS) [11,12]. The biological activity of Asta is usually related to the reduction of oxidative damage markers, and the production of inflammation is strongly related to oxidative stress. More and more studies have proven that Asta has sound anti-inflammatory effects [13]. Relevant studies have shown that F-Asta could effectively treat enteritis [14]. F-Asta could block the translocation of NF-κB into the nucleus of mucosal epithelial cells, suppress mucosal activation of MAPKs [15], and further prevent the development of DSS-induced UC [16]. However, F-Asta presents low bioavailability, as do many poorly water-soluble carotenoids [17]. Therefore, astaxanthin succinate diester (Asta-SD) was synthesized from F-Asta and succinic anhydride, based on the esterification of hydroxyl groups at both ends of F-Asta with hydrophilic fatty acids or amphiphilic molecules (Figure 1A). Relevant studies in our laboratory showed that the stability and bioavailability of Asta-SD were significantly higher than that of F-Asta [18]. Asta-SD is considered hydrolyzed into F-Asta and then absorbed by enterocytes. Compared with F-Asta, it is preliminarily verified that the polarity of Asta-SD is increased, which makes it easier to incorporate into the cell membrane and thereby be absorbed in tissue. However, to our knowledge, there is currently no scientific evidence regarding the ability of Asta-SD in the prevention and treatment of UC, especially regarding the role of intestinal microbiota in regulating inflammatory cytokines. Thus, further analyses are still required to investigate the biological functions of Asta-SD.

Therefore, this study evaluates the effect of a new water-soluble Asta-SD on dextran-sodium-sulfate-induced (DSS-induced) UC and whether gut microbiota was partly involved in it. We detected the effects of Asta-SD on the apparent and colonic biochemical indexes of UC mice and identified the critical bacteria through 16S rRNA sequencing and bioinformatics analysis.

## 2. Results

### 2.1. Improvement Effect of Asta-SD on Inflammation Model of Zebrafish

**Figure 2:**
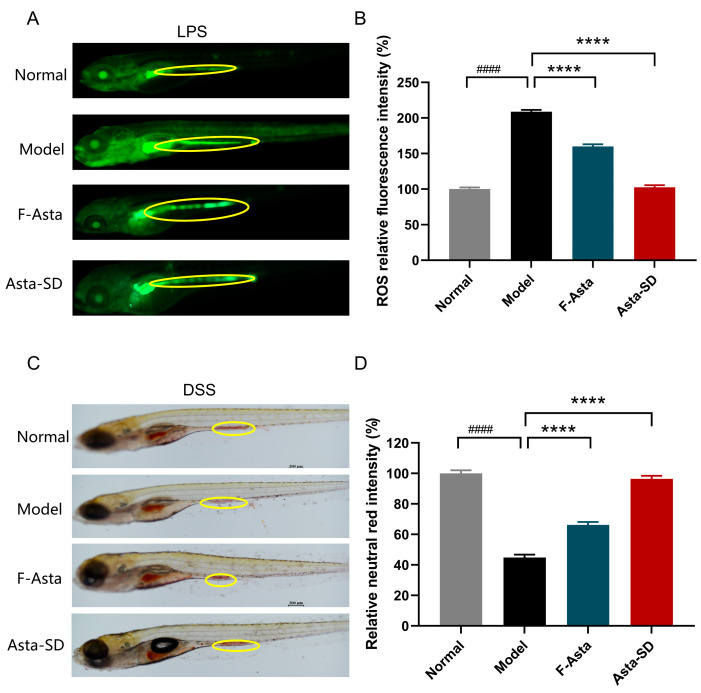
Inflammation inhibitory effect of astaxanthin succinate diester (Asta-SD) in zebrafish. (**A**) Quantification of the ROS levels induced by LPS, which were measured after staining with DCF-DA by image analysis and fluorescence microscopy. (**B**) Zebrafish fluorescence intensity using image J. (**C**) Quantification of neutral red images: injury in zebrafish intestines. (**D**) Neutral red accumulation. The values are expressed as the mean ± SEM, *n* = 10. #### *p* < 0.0001, **** *p* < 0.0001.

The zebrafish model was constructed to quickly evaluate the anti-inflammatory activity of Asta-SD against systemic inflammation stimulated by lipopolysaccharide (LPS) in vivo. LPS is a potent activator of gram-negative bacterial pathogens and innate immune responses [19]. It is widely used to investigate the acute inflammatory state in cells or tissues. The production of intracellular ROS was detected by using the oxidation-sensitive dye DCF-DA as a substrate. DCF-DA does not show fluorescence in the absence of ROS and becomes fluorescent when interacting with ROS [20], which is widely used to monitor the oxidative activity of cells. Therefore, the oxidation-sensitive fluorescent probe dye DCF-DA was used to analyze ROS production in the LPS-stimulated zebrafish inflammatory model. Zebrafish incubated with LPS showed a level of ROS production that was significantly higher than that of non-LPS treated zebrafish (Normal group). Treatment with Asta-SD significantly inhibited the level of ROS induced by LPS, indicating that Asta-SD significantly reduced the increase in ROS level caused by LPS treatment in the zebrafish model. This further confirmed the protective effect of Asta-SD on zebrafish inflammation (Figure 2A,B). Interestingly, in the zebrafish inflammation model, we noticed that Asta-SD significantly impacted the zebrafish’s visceral mass and intestine. Therefore, we further constructed the zebrafish intestinal inflammation model using DSS to investigate the improvement effect of Asta-SD.

We compared the effects of DSS injury in zebrafish to model inflammatory bowel disease (IBD) on the next immediate step. Similarly to DSS injury in mice, the intestinal length is significantly shortened in DSS-injured zebrafish. Neutral red is a dye that could diffuse through the cell membrane at a physiological pH (pH = 7) and could be significantly enriched in lysosome-rich intestinal epithelial cells (LREs). Related studies have shown that LREs could internalize dietary protein via receptor-mediated and fluid-phase endocytosis for intracellular digestion and trans-cellular transport [21,22]. The intensity of neutral red staining indicates the number of healthy lysosomes with acidic pH. A positive correlation between the number of healthy lysosomes and DSS-induced intestinal inflammation in zebrafish [23]. Therefore, we use the number of healthy lysosomes to observe the intestinal health of zebrafish. In the model group treated with DSS, the number of post-enteric lysosomes in zebrafish decreased sharply. In contrast, the number of lysosomes was restored effectively after treatment with Asta-SD (Figure 2C,D). Furthermore, Asta-SD has a specific biological safety effect according to the survival rate of zebrafish embryos in different concentrations of ASD at 48 h (the IC50 value: 5.75 g/L). Thus, it could be determined that the safety effect of Asta-SD could promote healthy acidified lysosomes and LREs in zebrafish and reduce DSS damage to the zebrafish intestinal tract by improving the number of lysosomes.

### 2.2. Influence on Body Weight and Colon Histology

**Figure 3:**
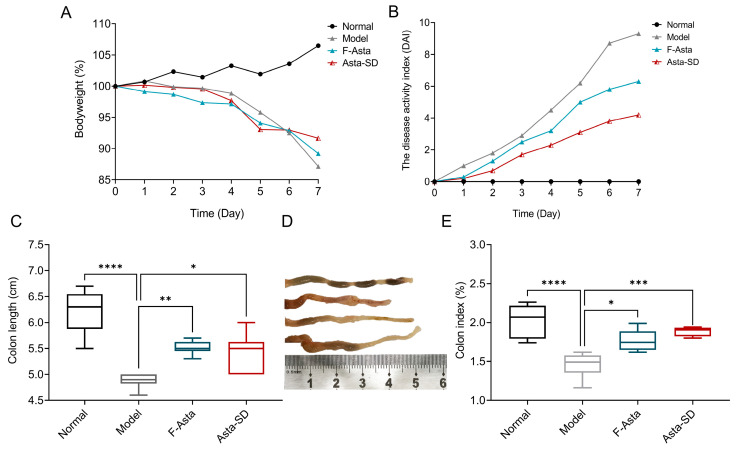
In vivo therapeutic outcomes of Asta-SD against UC. (**A**) Bodyweight in each group. (**B**) Kinetics of daily disease activity index (DAI) scores throughout the entire duration of the study. (**C**) Colon length. (**D**) Photographs of colons. (**E**) Colon index. Data were means ± SEM. (*n* = 6; * *p* < 0.05, ** *p* < 0.01, *** *p* < 0.001, and **** *p* < 0.0001; ANOVA followed by a Bonferroni post hoc test).

In this study, C57BL/6J mice under DSS-induced ulcerative colitis (UC) showed signs of severe weight loss. Therefore, during the 7-day oral administration, we focused on the weight change in the mice (as shown in Figure 3A). It was found that DSS intake resulted in a significant decrease in body weight (*p* ≤ 0.05). Compared with the Normal group, the colon length and weight of DSS-induced UC mice decreased significantly in the Model group (*p* < 0.01). The weight loss in C57BL/6J mice in the F-Asta and Asta-SD groups was slow, suggesting that Asta-SD might effectively alleviate the weight loss of mice with DSS-induced colitis.

To further delve into the effects of Asta-SD on the progress of UC, we calculated the disease activity index (DAI) of colitis by weight loss, diarrhea, and bloody stool in mice during the test [24]. Moreover, the ratio of colonic weight to body weight in UC mice was determined. Increased DAI scores (Figure 3B) and colonic shortening were observed (Figure 3C,D) in C57BL/6J mice with UC. However, Asta-SD administration remarkably decreased the DAI score and increased colon length in UC mice (*p* < 0.05, Figure 3B–D). Similarly, the reduction in colon index was reversed by Asta-SD treatment in mice with DSS-induced colitis (*p* < 0.05, Figure 3E).

### 2.3. Pathological Analysis

**Figure 4:**
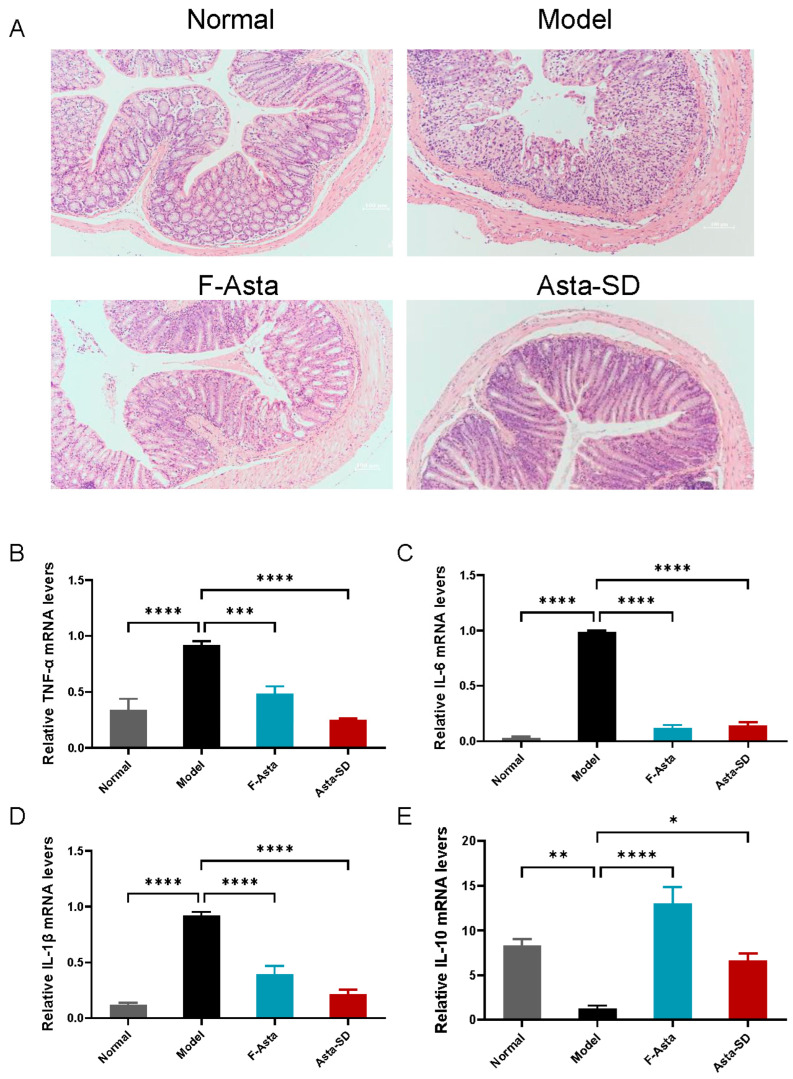
Astaxanthin succinate diester improves inflammation in UC mice. (**A**) Representative hematoxylin-and-eosin-stained (H&E-stained) distal colon sections. Gene expression levels of inflammatory factors: (**B**) TNF-α; (**C**) IL-6; (**D**) IL-1β; (**E**) IL-10. Data were means ± SEM. (*n* = 6; * *p* < 0.05, ** *p* < 0.01, *** *p* < 0.001, and **** *p* < 0.0001; ANOVA followed by a Bonferroni post hoc test).

We have performed an analysis of pathological changes to evaluate the effects of DSS and astaxanthin on colonic tissue. Studies have shown that the two mucus layers on the intestinal sidewall are essential to maintaining intestinal homeostasis. The inner mucus layer can be a potentially significant physical barrier for intestinal epithelial cells. As shown in Figure 4A, compared with the Normal group, the structures of the colonic crypt, epithelial cells, and goblet cells were seriously damaged in the Model group. Moreover, DSS treatment caused an apparent inflammation in the colon of the Model group. Through the observation of pathological sections, it can be observed that after the intervention of Asta-SD, the destruction of the intestinal barrier in the colon of DSS-induced UC mice was significantly improved. Namely, the intestinal mucosal damage has been effectively repaired, and the structure of goblet cells and crypts has remained intact.

### 2.4. Gene Expression of Inflammatory Factors in Colon Tissue

Once DSS destroys the colonic barrier function, it will trigger a series of inflammatory reactions in the colon tissue, which has a lot to do with the infiltration of inflammatory cells [25]. Increased cell infiltration in colitis may lead to excessive pro-inflammatory cytokines during the progression of colitis [26]. It was found that the level of IL-1β, IL-6, and TNF-α in the Model group was higher than that in the Normal group (based on the Normal group as units) but this significantly decreased after treatment with Asta-SD and F-Asta (*p* < 0.05, Figure 4B–E). At the same time, compared with the Normal group, DSS treatment significantly reduced the mRNA expression of IL-10 in the Model group (*p* < 0.5). After intervention with Asta-SD, the expression level of IL-10 increased significantly in DSS-treated mice (*p* < 0.5). Overall, these results showed that Asta-SD modulated the expression of inflammatory cytokines in UC mice.

### 2.5. Asta-SD Regulates Intestinal Microbial Structure and Composition

**Figure 5:**
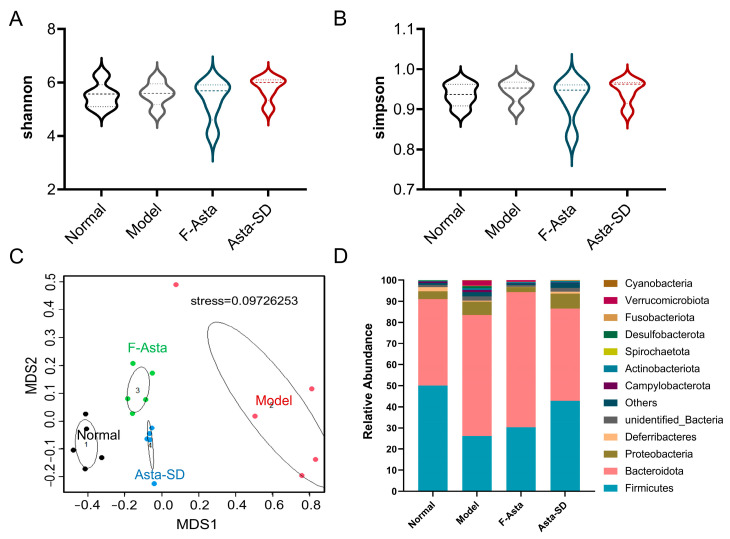
Asta-SD-regulated gut microbial structure and composition of DSS mice. The community diversity of gut microbiota (Shannon (**A**) and Simpson (**B**) index). (**C**) Nonmetric multidimensional scaling (NMDS) analysis based on Aitchison distance. (**D**) Bacterial taxonomic profiling at the phylum level.

In addition, 16S rRNA sequencing was performed to reveal whether Asta-SD affects intestinal microbiota. Firstly, representative sequences for each OTU were used to analyze Alpha diversity, such as Shannon and Simpson indexes [27]. As shown in Figure 5A,B, there was no significant difference in intestinal microbial diversity between groups. NMDS analysis was performed to evaluate the impact of supplementing with Asta-SD on the β-diversity of intestinal microbiota in UC mice. As shown in Figure 5C, the intestinal microbiota communities of the Model group and Normal group were significantly separated. The F-Asta and Asta-SD test groups were close but differed from the Model group and clustered independently. *Firmicutes* and *Bacteroidetes* were the most abundant taxa at the phylum level, followed by *Actinobacteria* (Figure 5D). Moreover, Asta-SD intervention could effectively reverse the reduction in the proportion of *Firmicutes*. Specifically, the ratio of *Firmicutes* to *Bacteroidetes* in the Asta-SD group is significantly higher than that of the Model group (*p* < 0.05).

**Figure 6:**
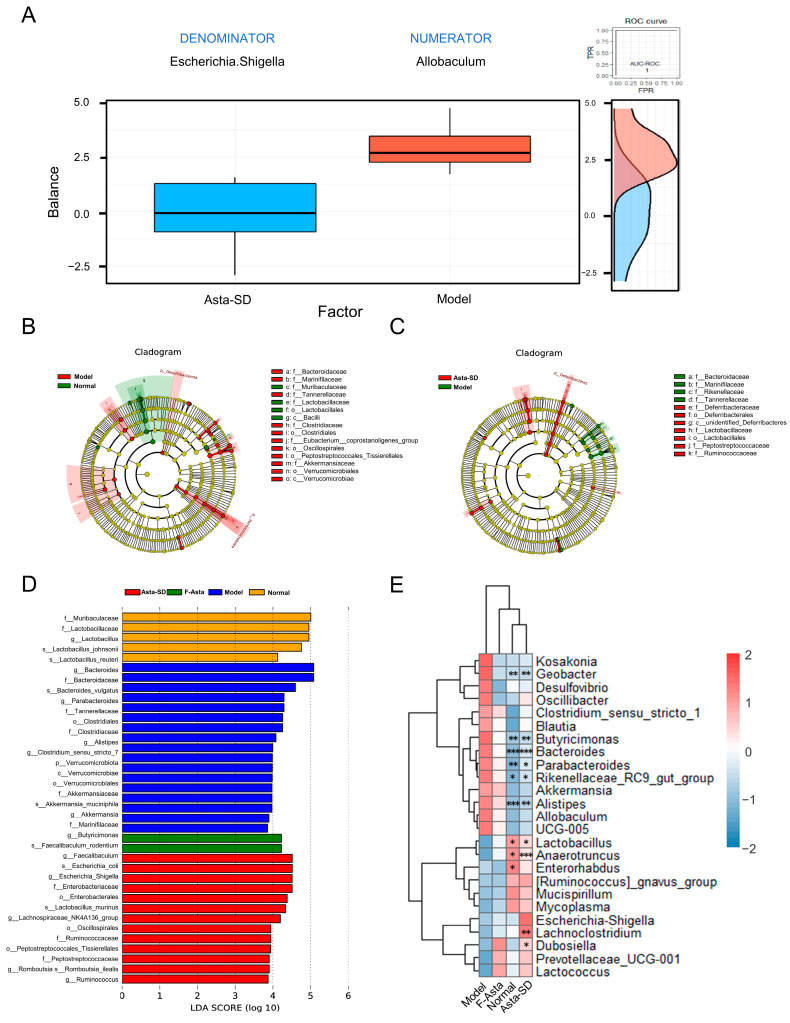
Asta-SD-regulated gut microbial structure and composition of UC mice. (**A**) The boxplot shows the distribution of the balance scores for the Model and Asta-SD groups. The ROC curve and the density plot are presented on the right side. (**B**,**C**) LEfSe evolutionary branching diagram. (**D**) Histogram of LDA value distribution (LDA > 3.5). (**E**) The heatmap illustrating gut microbial changes at the genus level for Normal and Asta-SD groups compared with the Model group (* *p* < 0.05, ** *p* < 0.01, *** *p* < 0.001).

To further analyze the important microbial characteristics related to the improvement of Asta-SD on DSS-induced ulcerative intestinal inflammation, Selbal balance analysis showed that *Escherichia Shigella* and *Allobaculum* had high prediction accuracy in predicting intestinal microbial changes caused by Asta-SD intervention in UC mice (AUC = 1, Figure 6A). Lefse analysis based on LDA (Figure 6B–D) found that Bacteroides and Parabacteroides in the Model group increased significantly compared with the Normal group. After the Asta-SD intervention, the microbiota composition in UC mice was substantially different from that in the Model group. It tended to be similar to that of the Normal group. In conclusion, Asta-SD could significantly change the structure and composition of intestinal microbiota in DSS-induced UC mice.

To further reveal the specific genera of intestinal flora affected by Asta-SD, the significantly different taxa were analyzed by MetaStat analysis (Figure 6E). Compared with the Normal group, the destruction of intestinal microbiota in the Model group was characterized by significant changes in the abundance of *Bacteroides*, *Alistipes*, *Geobacter*, *Butyrimionas*, *Parabacteroides*, *Anaerotruncus*, *Lactobacillus*, and *Enterhabdus* (*p* < 0.05). In addition, compared with the Model group, Asta-SD intervention significantly reduced the abundance of *Bacteroides*, *Geobacter*, *Alistipes*, *Butyrimionas*, and *Parabacteroides*. It significantly promoted the proliferation of *Anaerotruncus* and *Lactobacillus* (Figure 6E) (*p* < 0.05). The above results showed that Asta-SD positively affected the ecological imbalance of intestinal microbiota in mice with UC induced by 3% (*w*/*v*) DSS administration.

### 2.6. Asta-SD Affects the Correlation of the Intestinal Microbe and Anti-Inflammatory Factors in Mice with UC

**Figure 7:**
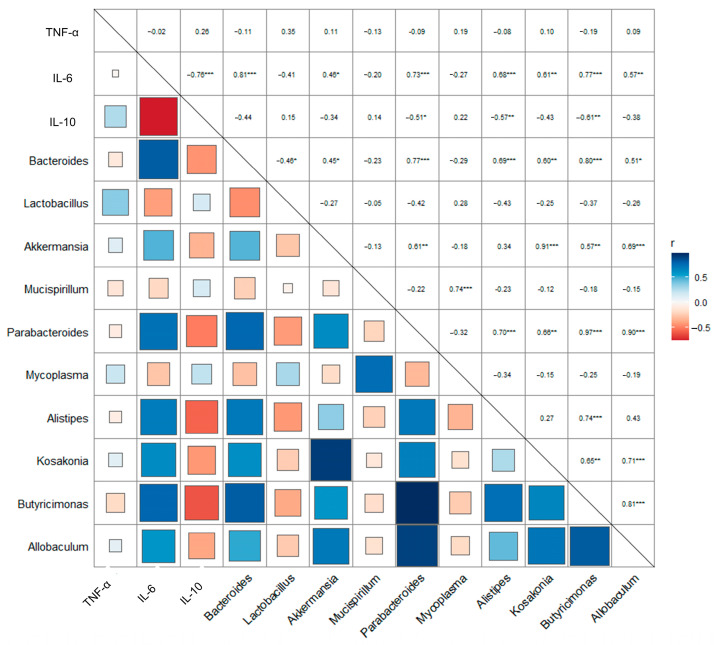
Pearson correlation analysis between pro-inflammatory cytokines and microbial communities at the genus. (* *p* < 0.05, ** *p* < 0.01, *** *p* < 0.001).

There is an interactive relationship between the microbiota and its biochemical indexes [28]. Pearson correlation analysis explores in depth the correlation between intestinal microbiota (at genus level) and inflammatory factor expression (Figure 7). The results showed that the gene expression of pro-inflammatory IL-6 was significantly negatively correlated with anti-inflammatory IL-10. Furthermore, *Bacteroides, Parabacteroides*, and *Butyrimionas* were significantly positively correlated with the gene expression of pro-inflammatory IL-6 (r > 0.7, *p* < 0.001).

## 3. Discussion

Astaxanthin has excellent antioxidant activity. The highly water-soluble Asta-SD based on free astaxanthin showed better solubility and antioxidant effects. In the in vivo anti-inflammation test model, the zebrafish is widely accepted as the best species for effective anti-inflammation assay because it has a well-developed innate and acquired immune system similar to the mammalian immune system [29]. Treating the systemic inflammation of zebrafish induced by LPS with Asta-SD could effectively eliminate the inflammation in zebrafish, especially in the digestive system. Therefore, we followed the zebrafish colitis model induced by DSS and found that Asta-SD did have an excellent diminishing zebrafish colitis effect, which is closely related to the significant antioxidant activity of Asta-SD.

Dietary astaxanthin has the potential to inhibit the occurrence of colitis [30]. In the present work, the effect of Asta-SD on DSS-induced UC in mice was explored first. DSS could directly damage intestinal epithelial cells and destroy the integrity of the mucosal barrier, resulting in the possibility of lumen bacteria entering the lamina propria and activating macrophages to secrete pro-inflammatory cytokines [31]. UC mice are induced by manifest with anorexia nervosa, weight loss, diarrhea, bloody stool, and colon shortening. Compared with the healthy controls, the colon length and body weight decreased significantly in DSS-induced UC mice (*p* < 0.01). Asta-SD’s intervention could effectively prevent the change in colon length and weight loss in mice exposed to DSS treatment.

Asta-SD’s effects on UC were further investigated by detecting the expression of inflammatory factors in mice. Observing pathological sections, we found that colonic mucosal injury in UC mice was effectively alleviated by Asta-SD intervention. Increased cell infiltration may lead to excessive production of pro-inflammatory cytokines during the progression of colitis [26]. This study confirmed that DSS could enhance the production of pro-inflammatory factors (IL-6, IL-1β, and TNF-α) and repress the secretion of anti-inflammatory IL-10. At the same time, Asta-SD could reverse the expression of these inflammatory factors to normal levels. These pro-inflammatory cytokines are typical features of DSS-induced colitis, since they can further mediate intestinal inflammation [5,32]. The expression of IL-6 and TNF-α has been reported to be mediated by intracellular signal transduction involving the NF-κB pathway and the activation of MAPKs [33]. Thus, we assume that Asta-SD might exert anti-inflammatory activity through the inhibition of the NF-κB p65/MAPK signaling pathway, followed by reduced expression of pro-inflammatory cytokines. In short, Asta-SD effectively ameliorated UC, improving inflammatory cell infiltration, inhibiting the expression of pro-inflammatory cytokines, and restoring intestinal barrier function [24].

The imbalance of intestinal microbiota will affect host intestinal physiology and further regulate the production of pro-inflammatory cytokines, leading to damage to the mucosal barrier and the occurrence of colitis [34]. In this study, the intestinal microbiota was studied by 16S rRNA gene sequencing, and the improvement effect of host-microbial interaction on colitis was subsequently analyzed. *Firmicutes* are gram-positive bacteria that are crucial to host nutrition and metabolism. *Firmicutes* contribute to regulating the physiological functions of other tissues and organs. The *Firmicutes*/*Bacteroidetes* (F/B) ratio is widely considered to impact intestinal homeostasis significantly. A decrease in the F/B ratio is often observed in IBD. *Firmicutes* have anti-inflammatory effects while *Bacteroidetes* exhibit pro-inflammatory properties mainly due to the production of endotoxins and cytokines, leading to IBD [35]. In this study, the intervention of Asta-SD could significantly improve the reduction in the F/B ratio caused by DSS.

In addition, specific microbiota members are closely linked with mouse colonic inflammation. Hence, differently abundant taxa were identified by bioinformatics analysis. The abundance of *Allobaculum* in the Model group is higher than in the Asta-SD group. *Allobaculum* is thought to be involved in inflammatory processes, and its relative abundance is positively correlated with ileal RORγT levels and IL-17 levels [36]. *Allobaculum* was isolated from patients with UC, resulting in more severe colitis when exposed to DSS. It may be related to the secretion of a high level of IgA [37]. This indicates that the *Allobaculum* has immunogenicity in vivo and may play an essential role in developing colonic inflammation.

Compared with the Normal diet group, intestinal microbial dysbiosis in the Model group is characterized by increased expression of pathogenic bacteria (especially *Bacteroides*, *Parabacteroides*, and *Butyrimionas*) and positive correlation with pro-inflammatory factor IL-6, as well as a decreased abundance of probiotics (such as *Lactobacillus* and *Anaerotruncus*). *Lactobacillus* has also been shown to resist DSS-induced colitis [38]. It could inhibit pathogenic bacteria by competing for nutrients and intestinal adhesion sites, inhibiting pathogenic bacteria, preventing cell apoptosis, and enhancing intestinal barrier function. Moreover, *Lactobacillus* prevents cell apoptosis in gut epithelium and improves intestinal barrier function [39]. *Lactobacillus* and *Anaerotruncus*, as probiotics, have been proven to effectively prevent and treat the occurrence of UC and inhibit its severe development via fecal microbiota transplantation (FMT) [40,41].

*Parabactoids* are considered to be dominant in the intestinal bacterial community related to the pathogenesis of UC [42]. Its enrichment in patients with inflammatory bowel disease or mouse models can lead to more severe symptoms [43]. Recent research has reported that Bacteroides play a vital role in initiating colitis [44]. This may be due to the increasing production of mucin-degrading sulphatases by Bacteroides. Mucin is an essential component of the intestinal mucosa. *Butyrimionas* are related to the occurrence of immune disorders [45], which are closely associated with LPS and D-lactic acid [46]. Notably, these two microbial metabolites are potential features of DSS-induced colitis [47]. In addition, our results do not rule out the possibility that changes in the abundance of other bacterial species will also affect the susceptibility of mice to UC. Therefore, specific genera (*Allobaculum*, *Bacteroides*, *Parabacteroides*, and *Butyrimionas*) associated with the expression of inflammatory factors might have the potential to become the therapeutic target of DSS-induced UC mice.

## 4. Materials and Methods

### 4.1. Materials

F-Asta (all-trans, 95.8 ± 0.5%) was purchased from Dr. Ehrenstorfer Co. (Augsbury, Germany). Asta-SD (>90%) was prepared in our laboratory. The 3-Aminophenylboronic acid (PBA) and 1-(3-Dimethylaminopropyl)-3-ethyl carbodiimide hydrochloride (EDC·HCl). DSS (MW: 36–40 kDa) was obtained from MP Biologicals (Solon, OH, USA).

### 4.2. Preparation and Purification of Asta-SD

The preparation and purification of Asta-SD were undertaken according to the method of Qiao et al. [18]. To prepare the F-Asta (1 mmol) and succinic anhydride (10 mmol) solution, weigh F-Asta and succinic anhydride separately with an analytical balance and dissolve them in 50 mL dioxane media. The reaction was catalyzed by triethylamine (3 mmol) at 55 °C. After 15 h, the reaction mixture was precipitated with 0.1 M hydrochloric acid (HCl) solution. The crude Asta-SD was purified using silica gel column chromatography for gradient elution using chloroform-methanol (100:0/97:3/90:10/0:100, *v*/*v*). Furthermore, the Asta-SD was collected and evaporated to dryness with a rotary evaporator at 25 °C to yield a highly purified Asta-SD. The purity and concentration of Asta-SD were determined by high-performance liquid chromatography with diode array detector, atmospheric pressure chemical ionization, and tandem mass spectrometry (HPLC-DAD-APCI-MS/MS), as reported earlier [18].

### 4.3. Intracellular Reactive Oxygen Species (ROS) in LPS-Stimulated Zebrafish

The wild-type zebrafish (Danio rerio) used in this study were cultured. The fertilized eggs were collected and raised at 28.5 °C in an incubator. The larvae were anesthetized with 0.02% Tricaine before fluorescent staining.

Synchronized zebrafish embryos were collected and arrayed by pipette, 15 embryos/well, in 12-well plates containing 2 mL embryo medium for 7–9 h post-fertilization (hpf). Fish were segregated into 4 groups: untreated (namely: Normal), LPS treated (namely: Model), F-Asta-, and Asta-SD- (astaxanthin equivalent is 25 μg/mL) treated groups (*n* = 10). To induce inflammation, the embryos were exposed to 10 µg/mL LPS dissolved in the embryo medium at 72 hpf at 28.5 °C. The intracellular ROS in zebrafish embryos was estimated using previously reported methods [19]. After 72 hpf, the zebrafish embryos were transferred into 24-well plates and separately stained with specific fluorescent probe dyes to determine intracellular ROS (2′,7′-dichlorodihydrofluorescein diacetate (DCF-DA). Following incubation for a specified period in the dye-containing medium, the embryos were rinsed with fresh embryo medium and anesthetized before observation, then observed under a fluorescence microscope equipped with a Nikon Eclipse Ti2 microscope (Nikon Instruments, New York, NY, USA). The images of the stained embryos were analyzed for intracellular ROS, and the fluorescence intensities of individual embryos were quantified using ImageJ software (ImageJ2).

### 4.4. Single DSS Injury Zebrafish Model

The single-injury protocol was adapted and modified by Oehlers et al. [48]. Batches of 60 larvae were segregated into Petri dishes in 50 mL of fresh embryo medium. Phenylthiourea (75 μM; Acros Organics, Geel, Belgium) was added at 24 hpf to prevent pigment-cell formation. To induce intestinal injury, 3 days post-fertilization (dpf) larvae were placed in freshly prepared 0.1 or 0.25% (*w*/*v*) colitis grade DSS (36,000–50,000 MW, MP Biomedical, Illkrich, France) for 3 days. Fish were segregated into 4 groups: untreated, DSS treated, F-Asta, and Asta-SD. Zebrafish larvae were stained live with 2.5 μg/mL Neutral Red (ACROS, Bridgewater, NJ, USA) in egg water for 5 h. The intensity of Neutral Red staining is non-saturated and consistent between 4 and 6 h. The images of the stained embryos were analyzed for Neutral Red staining with a Nikon Eclipse Ti2 microscope (Nikon Instruments, New York, NY, USA), and the fluorescence intensities of individual embryos were quantified using ImageJ software.

### 4.5. Animals and Treatments

All animal experiments were approved by the Committee on the Ethics of Animal Experiments of the College of Food Science and Engineering of the Ocean University of China (Certificate No. SPXY2022041601). Animal welfare was evaluated according to the Laboratory Animal Guideline for the ethical review of animal welfare. Five- to six-week-old specific pathogen-free (SPF) male C57BL/6 J mice were provided by Beijing Vital River Laboratory Animal Technology Co., Ltd. (Beijing, China). The mice were maintained at 24 ± 1 °C on a 12-h light–dark cycle. Upon arrival, the mice were acclimated for one week and fed with a standard diet (AIN-93M).

### 4.6. Sample Collection

The experimental design is shown in Figure 1. Animals were provided with free access to tap water supplemented with 3% DSS for seven days, followed by daily oral administration of 100 μL PBS in the presence or absence of 50 mg/kg body weight of F-Asta and Asta-SD. After one week of acclimation, mice were randomly divided into four groups (*n* = 6): the Normal diet group, the Model group (DSS-induced ulcerative colitis), the Asta-SD group, and the F-Asta group.

### 4.7. Evaluation of Disease Activity Index (DAI) Parameters and Colonic Damage

The body weight and physical state of mice were recorded daily for the entire duration of the study. Stool consistency and rectal bleeding were evaluated from the second week. The disease activity index (DAI) was evaluated to assess the severity of the colitis by combining scores of body weight loss, diarrhea of the stool, and the extent of blood in the feces, according to Peng et al. [24].

The gross morphological damage to the colonic mucosal membrane was also evaluated. Colon damage scores were assessed using the comprehensive score method. For future research, the remaining colon tissue was washed with PBS and snap-frozen in liquid nitrogen.

### 4.8. RT-qPCR Analysis of Inflammatory Cytokine Expression

Total RNA was extracted from the colon using Trizol (Invitrogen, Waltham, MA, USA). Then, cDNA was generated using a PrimeScript^®^ RT-PCR Kit (TaKaRa, DRR014A). Moreover, real-time PCR was performed using an IQTM5 Multicolor Real-Time PCR Detection System (Bio-Rad, Hercules, CA, USA). The relative gene expression change was calculated using the comparative method by normalization to the internal control, GAPDH. The sequences of all cytokine primers for PCR are listed in Table S1.

### 4.9. Determination of Colon Histology

The fresh colon tissues were fixed in paraformaldehyde (4% with PBS) for at least 36 h and then embedded in paraffin. The colon paraffin was cut into 5 μm slices and stained with hematoxylin and eosin. An inverted microscope was used to view colon pathology.

### 4.10. DNA Extraction, 16S rRNA Gene Sequencing, and Microbiota Data Analysis

Total microbial genomic DNA from samples was extracted using the CTAB method. DNA concentration and purity were monitored on 1% agarose gel via electrophoresis. Diluted DNA (1.0 ng/mL) was then used to amplify the V4-V5 region of the 16S rRNA gene using the primers 515F/926R and Phusion^®^ High-Fidelity PCR Master Mix with GC Buffer (New England Biolabs, Ipswich, MA, USA). Then, the amplicons were purified using Agencourt AMPure beads (Beckman Coulter Genomics, Brea, CA, USA) and quantified with a fluorescent-stain-based kit (Quant-iT PicoGreen, Invitrogen, Carlsbad, CA, USA).

Then, cleaned amplicons were sequenced on the Illumina HiSeq platform. Raw sequences were processed using the QIIME software (QIIME 2) package [49]. OTUs were picked at 97% similarity against the Silva database. Determinations of alpha and beta diversities were also conducted in QIIME. Significantly changed taxa between groups were identified by Linear Discriminant Analysis Effect Size (LEfSe) (Version 1.0, linear discriminant analysis [LDA] ≥ 3, *p* < 0.05). Most of the subsequent analyses were performed using R (version 4.1.0). In brief, the Vegan package calculated non-metric multidimensional scaling (NMDS). Balance analysis was conducted with the Selbal package. Pearson correlation analysis was completed using Vegan and Corrplot packages.

### 4.11. Statistical Analysis

All data were represented as means ± SEM and analyzed using the GraphPad Prism 8.0 program (GraphPad Software, San Diego, CA, USA). Data from more than two groups were compared using one-way ANOVA followed by Tukey’s multiple comparison tests. The adjusted *p* below 0.05 was considered statistically significant.

## 5. Conclusions

In conclusion, the new water-soluble Asta-SD has a better ameliorative effect on DSS-induced UC than F-Asta. Asta-SD maintains the intestinal microecological balance by regulating intestinal microbiota composition, repressing the growth of pathogenic bacteria, especially Bacteroides and Parabacteroides, and promoting the proliferation of beneficial bacteria (*Lactobacillus*, *Anaerotruncus*). Gut microbiota may partly contribute to the beneficial effects of Asta-SD on intestinal immunoregulation and damage repair. This study also supports Asta-SD as an alternative therapeutic agent for treating intestinal diseases, especially colitis. In addition, Asta-SD could be used as an intermediate to provide carboxyl groups for grafting water-soluble functional groups. It could also synthesize other astaxanthin derivatives with high water solubility and bioavailability.

## Data Availability

Not applicable.

## Supplementary Materials

The following supporting information can be downloaded at: https://www.mdpi.com/article/10.3390/molecules28186513/s1, Figure S1: The survival rate of zebrafish embryos in different concentrations of Asta-SD at 48 h; Table S1: Primer sequences used for qRT-PCR analysis.

## References

[1] Sandborn W.J., Su C., Sands B.E., D’Haens G.R., Vermeire S., Schreiber S., Danese S., Feagan B.G., Reinisch W., Niezychowski W. (2017). Tofacitinib as induction and maintenance therapy for ulcerative colitis. N. Engl. J. Med..

[2] Morgan X.C., Tickle T.L., Sokol H., Gevers D., Devaney K.L., Ward D.V., Reyes J.A., Shah S.A., Leleiko N., Snapper S.B. (2012). Dysfunction of the intestinal microbiome in inflammatory bowel disease and treatment. Genome Biol..

[3] Hu Q., Yuan B., Wu X., Du H., Gu M., Han Y., Yang W. (2019). Dietary intake of *Pleurotus eryngii* ameliorated dextran-sodium-sulfate-induced colitis in mice. Mol. Nutr. Food Res..

[4] Ge H., Cai Z., Chai J., Liu J., Liu B., Yu Y., Liu J., Zhang T. (2021). Egg White peptides ameliorate dextran sulfate sodium-induced acute colitis symptoms by inhibiting the production of pro-inflammatory cytokines and modulation of gut microbiota composition. Food Chem..

[5] Kanwal S., Joseph T.P., Aliya S., Song S., Saleem M.Z., Nisar M.A., Wang Y., Meyiah A., Ma Y., Xin Y. (2020). Attenuation of DSS induced colitis by Dictyophora Indusiata Polysaccharide (DIP) via modulation of gut microbiota and inflammatory related signaling pathways. J. Funct. Foods.

[6] De Almeida C.V., de Camargo M.R., Russo E., Amedei A. (2019). Role of diet and gut microbiota on colorectal cancer immunomodulation. World J. Gastroenterol..

[7] Mantovani A., Allavena P., Sica A., Balkwill F. (2008). Cancer-related inflammation. Nature.

[8] Hu T., Lin Q., Guo T., Yang T., Zhou W., Deng X., Yan J.-K., Luo Y., Ju M., Luo F. (2018). Polysaccharide isolated from *Phellinus linteus* mycelia exerts anti-inflammatory effects via MAPK and PPAR signaling pathways. Carbohydr. Polym..

[9] Oh N.S., Lee J.Y., Kim Y.-T., Kim S.H., Lee J.-H. (2020). Cancer-protective effect of a synbiotic combination between *Lactobacillus gasseri* 505 and a *Cudrania tricuspidata* leaf extract on colitis-associated colorectal cancer. Gut Microbes.

[10] Yadav V., Varum F., Bravo R., Furrer E., Bojic D., Basit A.W. (2016). Inflammatory bowel disease: Exploring gut pathophysiology for novel therapeutic targets. Transl. Res..

[11] Schönbeck C., Madsen T.L., Peters G.H., Holm R., Loftsson T. (2017). Soluble 1:1 complexes and insoluble 3:2 complexes—Understanding the phase-solubility diagram of hydrocortisone and γ-cyclodextrin. Int. J. Pharm..

[12] Wang H., He W., Mahukpégo Dansou D., Zhang H., Dwi Nugroho R., Tang C., Guo X., Yu Y., Zhao Q., Qin Y. (2022). Astaxanthin improved the storage stability of docosahexaenoic acid-enriched eggs by inhibiting oxidation of non-esterified poly-unsaturated fatty Acids. Food Chem..

[13] Kishimoto Y., Yoshida H., Kondo K. (2016). Potential anti-atherosclerotic properties of astaxanthin. Mar. Drugs.

[14] Zhang C., Xu Y., Wu S., Zheng W., Song S., Ai C. (2022). Fabrication of astaxanthin-enriched colon-targeted alginate microspheres and its beneficial effect on dextran sulfate sodium-induced ulcerative colitis in mice. Int. J. Biol. Macromol..

[15] Sakai S., Nishida A., Ohno M., Inatomi O., Bamba S., Sugimoto M., Kawahara M., Andoh A. (2019). Astaxanthin, a xanthophyll carotenoid, prevents development of dextran sulphate sodium-induced murine colitis. J. Clin. Biochem. Nutr..

[16] Chen Y., Su W., Tie S., Cui W., Yu X., Zhang L., Hua Z., Tan M. (2023). Orally deliverable sequence-targeted astaxanthin nanoparticles for colitis alleviation. Biomaterials.

[17] Ambati R.R., Moi P.S., Ravi S., Aswathanarayana R.G. (2014). Astaxanthin: Sources, extraction, stability, biological activities and its commercial applications—A review. Mar. Drugs.

[18] Qiao X., Yang L., Zhang T., Zhou Q., Wang Y., Xu J., Xue C. (2018). Synthesis, stability and bioavailability of astaxanthin succinate diester. J. Sci. Food Agric..

[19] Lee S.-H., Ko C.-I., Jee Y., Jeong Y., Kim M., Kim J.-S., Jeon Y.-J. (2013). Anti-Inflammatory effect of fucoidan extracted from *Ecklonia cava* in zebrafish model. Carbohydr. Polym..

[20] Handa O., Kokura S., Adachi S., Takagi T., Naito Y., Tanigawa T., Yoshida N., Yoshikawa T. (2006). Methylparaben potentiates UV-induced damage of skin keratinocytes. Toxicology.

[21] Park J., Levic D.S., Sumigray K.D., Bagwell J., Eroglu O., Block C.L., Eroglu C., Barry R., Lickwar C.R., Rawls J.F. (2019). Lysosome-rich enterocytes mediate protein absorption in the vertebrate gut. Dev. Cell.

[22] Tao P., Zhang B., Lin J., Wang S. (2021). Thrombospondin-1 aggravates colonic mucosal inflammatory injuries via promoting the differentiation of CD11c^+^ macrophages with lysosomal activity limited in colitis. Ann. Transl. Med..

[23] Chuang L., Morrison J., Hsu N., Labrias P.R., Nayar S., Chen E., Villaverde N., Facey J.A., Boschetti G., Giri M. (2019). Zebrafish modeling of intestinal injury, bacterial exposures and medications defines epithelial in vivo responses relevant to human inflammatory bowel disease. Dis. Model. Mech..

[24] Peng Y., Yan Y., Wan P., Chen D., Ding Y., Ran L., Mi J., Lu L., Zhang Z., Li X. (2019). Gut microbiota modulation and anti-inflammatory properties of anthocyanins from the fruits of *Lycium ruthenicum* Murray in dextran sodium sulfate-induced colitis in mice. Free Radic. Biol. Med..

[25] Tessner T.G., Cohn S.M., Schloemann S., Stenson W.F. (1998). Prostaglandins prevent decreased epithelial cell proliferation associated with dextran sodium sulfate injury in mice. Gastroenterology.

[26] Bian X., Wu W., Yang L., Lv L., Wang Q., Li Y., Ye J., Fang D., Wu J., Jiang X. (2019). Administration of *Akkermansia muciniphila* ameliorates dextran sulfate sodium-induced ulcerative colitis in mice. Front. Microbiol..

[27] Barger K., Langsetmo L., Orwoll E.S., Lustgarten M.S. (2020). Investigation of the diet-gut-muscle axis in the osteoporotic fractures in men study. J. Nutr. Health Aging.

[28] Hou D., Zhao Q., Yousaf L., Xue Y., Shen Q. (2020). Whole mung bean (*Vigna radiata* L.) supplementation prevents high-fat diet-induced obesity and disorders in a lipid profile and modulates gut microbiota in mice. Eur. J. Nutr..

[29] Sireswar S., Dey G., Biswas S. (2021). Influence of fruit-based beverages on efficacy of *Lacticaseibacillus rhamnosus* GG (*Lactobacillus rhamnosus* GG) against DSS-induced intestinal inflammation. Food Res. Int..

[30] Kohandel Z., Farkhondeh T., Aschner M., Pourbagher-Shahri A.M., Samarghandian S. (2022). Anti-inflammatory action of astaxanthin and its use in the treatment of various diseases. Biomed. Pharmacother..

[31] Leiba J., Özbilgiç R., Hernández L., Demou M., Lutfalla G., Yatime L., Nguyen-Chi M. (2023). Molecular actors of inflammation and their signaling pathways: Mechanistic insights from zebrafish. Biology.

[32] Wang K., Jin X., Li Q., Sawaya A.C.H.F., Le Leu R.K., Conlon M.A., Wu L., Hu F. (2018). Propolis from different geographic origins decreases intestinal inflammation and *Bacteroides* spp. populations in a model of DSS-induced colitis. Mol. Nutr. Food Res..

[33] Nishida A., Hidaka K., Kanda T., Imaeda H., Shioya M., Inatomi O., Bamba S., Kitoh K., Sugimoto M., Andoh A. (2016). Increased expression of interleukin-36, a member of the interleukin-1 cytokine family, in inflammatory bowel disease. Inflamm. Bowel Dis..

[34] Cai X., Han Y., Gu M., Song M., Wu X., Li Z., Li F., Goulette T., Xiao H. (2019). Dietary cranberry suppressed colonic inflammation and alleviated gut microbiota dysbiosis in dextran sodium sulfate-treated mice. Food Funct..

[35] Stojanov S., Berlec A., Štrukelj B. (2020). The influence of probiotics on the firmicutes/bacteroidetes ratio in the treatment of obesity and inflammatory bowel disease. Microorganisms.

[36] Cox L.M., Yamanishi S., Sohn J., Alekseyenko A.V., Leung J.M., Cho I., Kim S.G., Li H., Gao Z., Mahana D. (2014). Altering the intestinal microbiota during a critical developmental window has lasting metabolic consequences. Cell.

[37] Palm N.W., de Zoete M.R., Cullen T.W., Barry N.A., Stefanowski J., Hao L., Degnan P.H., Hu J., Peter I., Zhang W. (2014). Immunoglobulin A coating identifies colitogenic bacteria in inflammatory bowel disease. Cell.

[38] Zhang F., Li Y., Wang X., Wang S., Bi D. (2019). The impact of lactobacillus plantarum on the gut microbiota of mice with DSS-induced colitis. BioMed Res. Int..

[39] Jiang D., Kang A., Yao W., Lou J., Zhang Q., Bao B., Cao Y., Yu S., Guo S., Zhang Y. (2018). Euphorbia kansui fry-baked with vinegar modulates gut microbiota and reduces intestinal toxicity in rats. J. Ethnopharmacol..

[40] Wen X., Wang H.-G., Zhang M.-N., Zhang M.-H., Wang H., Yang X.-Z. (2021). Fecal microbiota transplantation ameliorates experimental colitis via gut microbiota and T-cell modulation. World J. Gastroenterol..

[41] Hamamah S., Gheorghita R., Lobiuc A., Sirbu I.-O., Covasa M. (2022). Fecal microbiota transplantation in non-communicable diseases: Recent advances and protocols. Front. Med..

[42] Dziarski R., Park S.Y., Kashyap D.R., Dowd S.E., Gupta D. (2016). *Pglyrp*-regulated gut microflora *Prevotella falsenii*, *Parabacteroides distasonis* and *Bacteroides eggerthii* Enhance and *Alistipes finegoldii* attenuates colitis in mice. PLoS ONE.

[43] Bharadwaj R.S. (2016). Role of bacteria in Inflammatory Bowel Disease (IBD). Int. J. Infect. Dis..

[44] Lucke K., Miehlke S., Jacobs E., Schuppler M. (2006). Prevalence of *Bacteroides* and *Prevotella* spp. in ulcerative colitis. J. Med. Microbiol..

[45] Enemchukwu C.U., Ben-Faras H., Gialanella P., Szymczak W.A., Nosanchuk J.D., Madaline T.F. (2016). Butyricimonas virosa bacteraemia and bowel disease: Case report and review. New Microbes New Infect..

[46] Ulger Toprak N., Bozan T., Birkan Y., Isbir S., Soyletir G. (2015). Butyricimonas virosa: The first clinical case of bacteraemia. New Microbes New Infect..

[47] Zhu L., Song Y., Liu H., Wu M., Gong H., Lan H., Zheng X. (2021). Gut microbiota regulation and anti-inflammatory effect of β-carotene in dextran sulfate sodium-stimulated ulcerative colitis in rats. J. Food Sci..

[48] Oehlers S.H., Flores M.V., Hall C.J., Okuda K.S., Sison J.O., Crosier K.E., Crosier P.S. (2013). Chemically induced intestinal damage models in zebrafish larvae. Zebrafish.

[49] Bokulich N.A., Subramanian S., Faith J.J., Gevers D., Gordon J.I., Knight R., Mills D.A., Caporaso J.G. (2013). Quality-filtering vastly improves diversity estimates from illumina amplicon sequencing. Nat. Methods.

