# Supplementary material for: In-Depth Analysis of the Mechanism of Astaxanthin Succinate Diester in Reducing Ulcerative Colitis in C57BL/6J Mice Based on Microbiota Informatics
Source: Molecules. 2023 Sep 8;28(18):6513. doi: 10.3390/molecules28186513 (PMC10537600; doi:10.3390/molecules28186513)
Supplement: Supplementary file 1 [file molecules-28-06513-s001.zip › molecules-2577625-supplementary.pdf]

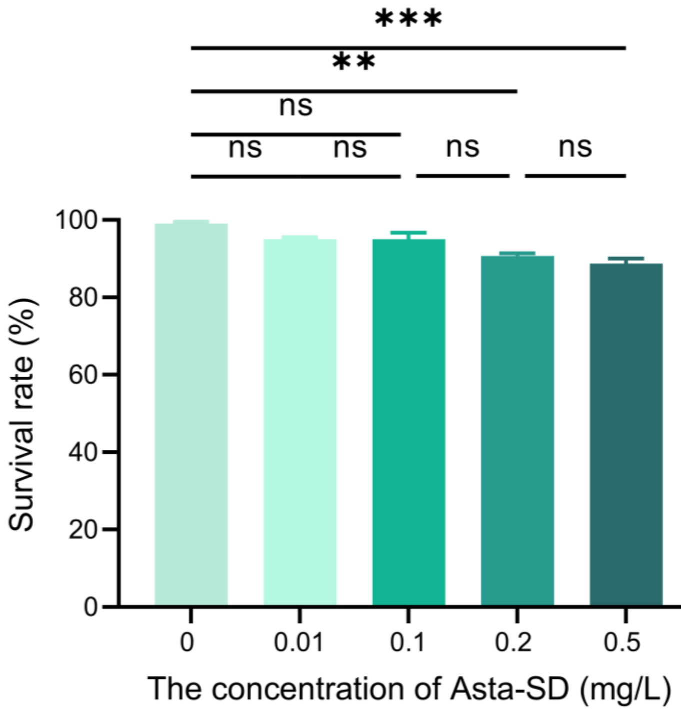

Figure S1. The survival rate of zebrafish embryos in different concentrations of Asta-SD at 48 hours. ($p < 0.01$ and *** p < 0.001; ANOVA followed by a Bonferroni post hoc test)**

Table S1. Primer Sequences Used for qRT-PCR Analysis

Primer	Forward primer	Reverse primer
IL-1β	ACTCATTGTGGCTGTGGAGA	TTGTTCATCTCGGAGCCTGT
IL-6	CTCTGGCGGAGCTATTGAGA	AAGTCTCCTGCGTGGAGAAA
TNF-α	CCCTCACACTCAGATCATCTTCT	CTACGACGTGGGCTACAG
IL-10	CAGAGAAGCATGGCCCAGAA	GCTCCACTGCCTTGCTCTTA
GAPDH	TGGAGAAACCTGCCAAGTATGA	TGGAAGAATGGGAGTTGCTGT
